# 肺腺癌靶向治疗后小细胞肺癌转化的病例报道

**DOI:** 10.3779/j.issn.1009-3419.2015.10.10

**Published:** 2015-10-20

**Authors:** 淑萍 薛, 婷婷 俞, 琰 张, 莉 单

**Affiliations:** 830000 乌鲁木齐，新疆医科大学附属肿瘤医院肺内一科 The First Department of Lung Cancer, Xinjiang Tumor Hospital, Urimuqi 830000, China

**Keywords:** 肺肿瘤, *EGFR*基因突变, 耐药, Lung neoplsms, *EGFR* mutation, Resistance

## Abstract

近年来, 非小细胞肺癌(non-small cell lung cancer, NSCLC)静脉化疗逐渐进入平台期, 缓解率(response rate, RR)、平均总生存期(overall survival, OS)无明显改善; 随着分子生物学发展, 以表皮生长因子受体(epidermal growth factor receptor, EGFR)为靶点的EGFR酪氨酸激酶抑制剂(EGFR-tyrosine kinase inhibitors, EGFR-TKIs)在NSCLC的治疗中疗效显著, 但患者最终均会出现耐药, 关于耐药机制目前尚未完全明确。本文详细报道1例49岁女性*EGFR*基因19外显子缺失突变肺腺癌患者, 在靶向治疗耐药后二次活检发现转化为小细胞肺癌(small cell lung cancer, SCLC), 予以SCLC化疗方案两周期有效; 通过分析患者诊疗过程、疗效, 对相关机制进展作一综述。

## Abstract (en)

In recent years, the chemotherapy of non-small cell lung cancer (NSCLC) has almost been reached a platform stage, and there is no obvious progress in terms of response rate (RR) and overall survival (OS); With the great development of molecular biology, epidermal growth factor receptor-tyrosine kinase inhibitors (EGFR-TKIs) has good therapeutic effect on the NSCLC.But, almost all patients of *EGFR*-mutant lung cancers develop drug resistance to these agents.This paper reported a case of a 49-year-old woman with lung adenocarcinoma who had *EGFR* mutant (19-DEL) treated with EGFR-TKIs.After disease progression, histological examination of a secondary biopsy specimen revealed small cell lung cancer (SCLC) had transformed to SCLC treatment.Through the analysis of the process and effect of her therapy, the following is a summary of the relevant mechanism.

肺癌目前仍是世界发病率及死亡率最高的恶性肿瘤^[1]^, 近年来随着肿瘤分子生物学的发展、“精准医学”观念的提出, 肺癌的治疗日趋个体化。针对分子靶点的新一代抗肿瘤药物将凭借其特异性和靶向性, 成为晚期肺癌的研究及治疗热点, 其中最具代表性的是以表皮生长因子受体(epidermal growth factor receptor, EGFR)为靶点的基因突变型肺腺癌患者的靶向治疗。EGFR是表皮生长因子受体家族成员之一, 具有酪氨酸激酶活性, 参与细胞中一系列重要的生理过程, 与肿瘤细胞的增殖、血管生成、肿瘤侵袭、转移有关, 在多种实体肿瘤中过表达^[2]^。EGFR酪氨酸激酶抑制剂(tyrosine kinase inhibitors, TKIs)可逆地与EGFR的三磷酸腺苷结合位点相竞争, 阻断EGFR信号传递, 从而抑制肿瘤生长、增殖^[3]^。国内外临床研究^[4-6]^已证实, 70% *EGFR*突变者对靶向药物有效, EGFR-TKIs一线治疗*EGFR*突变的晚期非小细胞肺癌(non-small cell lung cancer, NSCLC)疗效优于单纯化疗, 可延长患者无进展生存期(progression free survival, PFS)及总生存期, 美国国立综合癌症网络(National Comprehensive Cancer Network, NCCN)指南已将EGFR-TKI做为晚期*EGFR*突变肺腺癌患者一线治疗方案^[7]^。但令人遗憾的是随着用药时间的延长, 多数患者在平均用药12个月后出现病情进展。关于EGFR-TKI耐药机制目前发现最常见的为T790M突变, 约占50%;其余耐药机制包括C-Met、HER-2扩增(15%-20%)、PI3K旁路激活(< 5%)等^[8]^。在已知的耐药机制中存在一种虽少见, 却不容忽视的类型:小细胞肺癌转化(6%-14%)^[9-13]^, 现报道1例以小细胞肺癌转化为EGFR-TKIs耐药机制的肺腺癌。

## 1 临床病史

患者女性, 49岁, 汉族, 无吸烟史, 于2012年2月20日以" 咳嗽、咳痰2个月, 发现右肺占位10天" 为主诉就诊我院, 于2012年3月6日在全麻下行" 胸腔镜右肺下叶完全性切除术+纵隔淋巴结清扫术" 。术后病理:(右肺下叶)中分化腺癌。术后分期" 右肺中分化腺癌pT2N2M0 Ⅲa期" ,术后行3周期培美曲塞+顺铂行化疗; 因患者化疗期间消化道反应明显暂停术后化疗。2013年2月复查计算机断层扫描(computed tomography, CT)示:右肺癌术后改变, 右肺下叶支气管断端周围片状软组织影, 两侧锁骨区、纵隔、右肺门多发淋巴结(大者1.5 cm×2.3 cm), 提示术后复发。因术后复发病灶较小, 位于中央, 穿刺活检风险较大, 未能行二次活检。行原手术标本*EGFR*基因检测:19外显子缺失突变。于2013年5月开始口服吉非替尼250 mg, 1次/天, 服药1个月后复查CT, 疗效评价:部分缓解。2013年6月-2014年6月定期入院复查CT疗效评价:部分缓解。此期间胃泌素释放肽前体、神经元特异性烯醇化酶处于正常范围。2014年11复查CT示:左侧锁骨区、右肺门、纵隔淋巴结较前增大, 根据实体瘤疗效评价标准(Response Evaluation Criteria in Solid Tumors, RECIST), 增大未超过20%, 疗效评价为疾病稳定(stable disease, SD), 患方要求继续口服靶向药物。患者2015年3月因咳嗽咳痰伴右下肢疼痛, 胸腰部疼痛, 卡氏体能状态(Karnofsky performance status, KPS)评分60分, 就诊于外院, 完善相关检查(具体不详)考虑吉非替尼靶向后耐药, 于2015年3月中旬停止口服吉非替尼片, 2015年3月18日开始口服靶向药物AZD9291(由海外购入), 症状缓解不佳。2015年4月24日我院CT示:右肺下叶支气管断端周围软组织影, 较前增大, 左侧锁骨区、右肺门、纵隔结较前增大, 考虑转移瘤, 两肺转移瘤较前增大; 肝脏、脾脏、双侧肾上腺、双肾结节、肿块—考虑转移瘤; 多发骨转移瘤。提示靶向治疗疗效为疾病进展。同时胃泌素释放肽前体 > 5, 000.00 pg/mL, 神经元特异性烯醇化酶352.00 ng/mL, 较前明显增高, 气管镜示:右肺上叶癌, 病检示结合免疫组化结果(图 1):CK7(+)、CK5/6(-)、Ki-67(+, 80%)、TTF-1(+)、P63(-)、CD56(+)、Syn(+)、CgA(-), 提示小细胞肺癌。考虑肺腺癌靶向治疗后转化成小细胞肺癌, 诊断为:右肺恶性肿瘤(右肺上叶小细胞肺癌T4N3M1 Ⅳ期, 广泛期); 因患者化疗耐受性差, 遂于2015年4月29日行第1周期单药依托泊苷注射液化疗(依托泊苷注射液0.1 g, 静点, d1-d5), 第二周期于5月26日行方案" EC" (依托泊苷+卡铂)化疗, 具体剂量为(依托泊苷注射液0.1 g, d1-d5, 卡铂450 mg, d1)。两周期后患者诉胸腰部、右下肢疼痛较前明显缓解, KPS评分80分。2015年6月24日CT检查示:右肺下叶支气管断端周围软组织影, 较前缩小; 疗效评价SD(稳定中好转)。期间复查CT表现见图 2。

**1:**
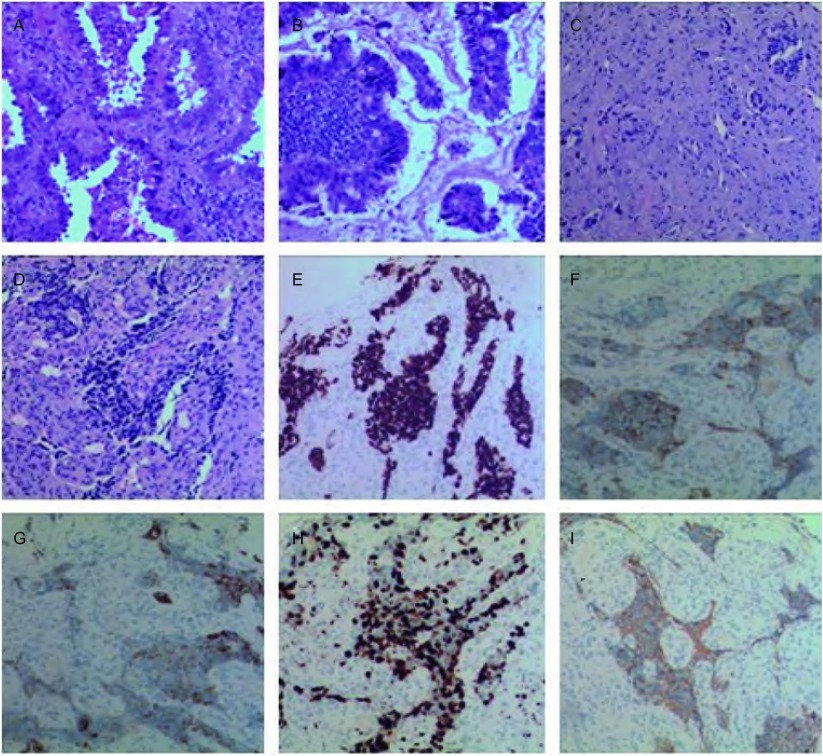
病理图片:A-B:患者诊断为初次病检肺腺癌HE染色; C-I:二次病检诊断为小细胞肺癌病理图片, C-D:HE染色图片, E-I:诊断为小细胞肺癌免疫组化图片(E:TIF-1;F:SYN; G:KI67;H:CK7;I:CD56)(SP, ×100)。 Pathological pictures:Hematoxylin-eosin staining of a primary biopsy specimen (A-B).Hematoxylin-eosin staining of a secondary biopsy specimen (C-D) that was positive for TIF-1 (E), synaptophysin (F), KI67 (G), CK7 (H) and CD56 (I) (SP, ×100).

**2:**
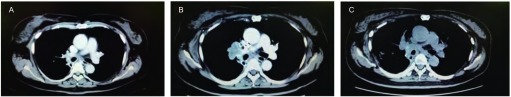
治疗期间胸部CT变化。A:吉非替尼治疗后1月(2013年6月); B:吉非替尼药后进展(2015年4月); C:周期小细胞化疗方案后(2015年6月)。 The changes of chest CT in different stages.A:Targeted therapy for a month (June, 2013);B:Resistance to Gefitinib (April, 2015);C:After two cycle SCLC chemotherapy (April, 2015).CT:computed tomography; SCLC:small cell lung cancer.

## 2 讨论

关于小细胞肺癌转化所致的耐药机制目前尚不完全明确, 在服用EGFR-TKIs后为何会出现不同病理类型的转换？EGFR-TKIs是导致肺腺癌中发现小细胞成分的唯一原因？早在1986年人们对最初诊断为肺腺癌的患者尸检时发现小细胞肺癌成分^[14]^; Sequist等^[8]^对靶向治疗后耐药*EGFR*基因突变型肺腺癌患者行二次活检, 发现病理组织转化为小细胞肺癌, 且转化后小细胞肺癌组织仍保持原有*EGFR*基因突变型, 提示他们可能来源于同一组织。但是更早的研究者在未行EGFR-TKIs治疗的*EGFR*突变型腺癌组织中也发现腺癌与小细胞肺癌两种成分^[15]^; 且在最初诊断为EGFR野生型腺癌患者中也发现了小细胞肺癌成分^[16]^。以上说明无论有无*EGFR*基因突变、是否行TKI治疗, 最初诊断为腺癌组织均可能出现小细胞肺癌成分; 目前认为可能存在两种可能^[17]^:①原始肿瘤即存在两种混合成分, 最初诊断的可能为优势主导成分; ②靶向药物治疗后诱发腺癌向小细胞肺癌转化。虽然*EGFR*基因突变型肺腺癌靶向治疗不是导致出现小细胞肺癌唯一的原因, 但目前认为*EGFR*基因突变型患者在接受EGFR-TKI治疗后转化为小细胞肺癌的几率较EGFR野生型高^[16, 17]^。

本文中此例肺腺癌患者经EGFR-TKIs靶向治疗有效1年后, 出现病情进展。患者为术后标本, 取材数量充足, 最初未能发现混合型可能性较小。令人遗憾的是复发后因取得病理组织困难, 未能行二次活检。虽不能病理证实术后复发时是否存在混合型, 但患者复发至靶向耐药期间, 对靶向药物治疗有效, 且胃泌素释放肽前体、神经元特异性烯醇化酶等小细胞相关肿瘤抗原靶向治疗前及有效期间均处于正常水平, 靶向治疗耐药后明显升高, 且肿块迅速增大、广泛远处转移, 符合小细胞肺癌生物学特性。服用靶向药物AZD9291无效, 提示T790M突变耐药可能性小。所以我们认为患者EGFR-TKIs耐药机制与非小细胞肺癌转化为小细胞肺癌有关。

虽然目前尚未明确导致接受靶向治疗的肺腺癌转化为小细胞肺癌, 但部分研究观点认为这种能够转化的*EGFR*突变型肺腺癌与小细胞肺癌之间可能在发病机制上有共同起源, 可能对今后明确转化机制提供启示。Rudin等^[9]^与Peifer等^[18]^通过老鼠动物模型基因测序发现, 肺腺癌中多见*EGFR*、*KRAS*基因突变, 而在小细胞肺癌中, 多见*RB1*及*Tp53*基因突变。*RB1*基因缺失突变在小细胞肺癌中几乎可达100%^[8]^。后来人们对肺腺癌转化为小细胞肺癌患者再次活检分析基因型发现100%患者肺癌组织中均存在*RB1*基因缺失^[19]^, 这暗示在此类转化耐药患者中RB1缺失可能在转化过程中发挥一定作用, 亦或EGFR-TKIs可能导致RB1缺失的出现。

另一个导致转化的原因可能与Ⅱ型肺泡上皮细胞的增殖与分化有关, 通常我们认为肺腺癌来源于肺泡上皮细胞^[20]^, 小细胞肺癌来源于神经内分泌细胞^[21]^; Sutherland等^[21]^发现当敲除Ⅱ型肺泡上皮细胞中的RB1及Tp53基因时, 会导致小细胞肺癌, Ⅱ型肺泡上皮细胞具有转化为小细胞肺癌及肺腺癌等潜能。Ⅱ型肺泡上皮细胞增殖及分化受EGFR水平调节^[22]^, EGFR表达可促进Ⅱ型肺泡上皮细胞增殖及分化, 当*EGFR*基因突变患者使用EGFR-TKIs后, 可能导致这种促进其分化的力量削弱, 甚至消失, 异常分化可能导致Ⅱ型肺泡上皮细胞向小细胞肺癌发展。以上这两种原因可能在*EGFR*突变型腺癌靶向治疗后向小细胞肺癌转化中发挥着一定作用, 但所占分量目前还不能明确。若条件允许对*EGFR*突变型肺腺癌患者行RB1基因检测, 可能成为一个预测肺腺癌耐药后向小细胞肺癌转化的指标。

虽然肺腺癌转化为小细胞肺癌机制尚不明确, 但目前此类转化型肺癌的治疗多以小细胞肺癌标准治疗为主^[23]^, 给予" 依托泊苷联合铂类" 方案化疗。本例患者对小细胞肺癌化疗方案敏感性似乎与原发小细胞肺癌不同, 一方面可能与患者第一周期未行标准" 依托泊苷联合铂类" 方案化疗有关; 另一方面, 转化型小细胞肺癌可能存在尚未明确的、与原发小细胞肺癌不同的病理机制, 需要后期大量的研究去发现明确。患者行两周期小细胞化疗方案后疗效评价为稳定中有好转, 虽未达到我们预期的明显缓解效果, 但患者行" EC" 方案(依托泊苷+卡铂)化疗后, 诉胸腰椎处疼痛较前明显缓解, KPS评分较前升高, 生活质量提高。目前患者继续" EC”化疗方案中。

为明确靶向治疗后肺腺癌转化为小细胞肺癌的原因, 还需要大量的临床及实验室研究。国际上虽有NSCLC转化为小细胞肺癌案例报道, 但在临床工作中类似病例很少见, 部分原因可能是多数耐药后病例未能行二次活检, 未发现病理类型的转化。对于靶向治疗后耐药的*EGFR*基因突变型患者, 均应进行二次活检。目前临床病理明确靶向耐药后转化型小细胞肺癌患者数量有限, 尚不能明确哪些临床特点可促使肺腺癌向小细胞肺癌转化。我们认为对于耐药后小细胞肺癌相关肿抗明显升高时或伴有*RB1*基因缺失者转化可能性较大。靶向治疗以基因突变点为靶标, 在反复治疗中, 患者的基因靶点可能出现变化。对于治疗后耐药患者积极行再次组织活检十分必要。明确患者基因型变化情况, 调整制定精确治疗方案, 才能真正达到个体化精准治疗目的。
